# Calibration of Low-Cost Moisture Sensors in a Biochar-Amended Sandy Loam Soil with Different Salinity Levels

**DOI:** 10.3390/s24185958

**Published:** 2024-09-13

**Authors:** María José Gómez-Astorga, Karolina Villagra-Mendoza, Federico Masís-Meléndez, Aníbal Ruíz-Barquero, Renato Rimolo-Donadio

**Affiliations:** 1 Agricultural Engineering, CETIA Centro de Investigación y Extensión en Tecnología e Ingeniería Agrícola, Instituto Tecnológico de Costa Rica, Cartago P.O. Box 159-7050, Costa Rica; mar.gomez@itcr.ac.cr; 2 Chemistry, CEQIATEC, Centro de Investigación y de Servicios Químicos y Microbiológicos, Instituto Tecnológico de Costa Rica, Cartago P.O. Box 159-7050, Costa Rica; fmasis@itcr.ac.cr; 3 Electronic Engineering, Instituto Tecnológico de Costa Rica, Cartago P.O. Box 159-7050, Costa Rica; aniruiz@itcr.ac.cr (A.R.-B.); rrimolo@itcr.ac.cr (R.R.-D.)

**Keywords:** calibration, soil moisture sensor, capacitive sensor, soil moisture content, salinity

## Abstract

With the increasing focus on irrigation management, it is crucial to consider cost-effective alternatives for soil water monitoring, such as multi-point monitoring with low-cost soil moisture sensors. This study assesses the accuracy and functionality of low-cost sensors in a sandy loam (SL) soil amended with biochar at rates of 15.6 and 31.2 tons/ha by calibrating the sensors in the presence of two nitrogen (N) and potassium (K) commercial fertilizers at three salinity levels (non/slightly/moderately) and six soil water contents. Sensors were calibrated across nine SL-soil combinations with biochar and N and K fertilizers, counting for 21 treatments. The best fit for soil water content calibration was obtained using polynomial equations, demonstrating reliability with $R^{2}$ values greater than 0.98 for each case. After a second calibration, low-cost soil moisture sensors provide acceptable results concerning previous calibration, especially for non- and slightly saline treatments and at soil moisture levels lower than 0.17 cm^3^cm^−3^. The results showed that at low frequencies, biochar and salinity increase the capacitance detected by the sensors, with calibration curves deviating up to 30% from the control sandy loam soil. Due to changes in the physical and chemical properties of soil resulting from biochar amendments and the conductive properties influenced by fertilization practices, it is required to conduct specific and continuous calibrations of soil water content sensor, leading to better agricultural management decisions.

## 1. Introduction

The growth in population is associated with an increase in food demand, reduced water availability, and a significant impact on agriculture [1]. Over time, significant efforts have been made to improve water use efficiency, based on the idea that it is possible to obtain greater results with less water through more efficient management [2].

Currently, the development of innovative systems for smart agriculture emphasizes the integration of different technologies [3], particularly since irrigation is one of the largest consumers of freshwater in the agricultural sector [4].

Numerous irrigation systems documented in the literature utilize soil moisture sensors to monitor soil moisture levels and activate irrigation accordingly [5,6,7].

However, the limitations of these sensors pose challenges in terms of accuracy and robustness; therefore, some studies have been conducted on sensor performance and comparison. One of the challenges is the scarcity of accurate calibration data for soil moisture sensors, which restricts the use of smart sensors in various commercial applications. Although the scientific literature provides several methods for calibrating dielectric soil moisture sensors, technical complexity, factors influencing calibration, and a lack of confidence in their accuracy limit their application in field studies [8].

These sensors require soil-specific calibration, both in the field and the laboratory, as there is often significant variability between sensors, which can affect the accuracy of measurements [9].

Another problem is the cost–accuracy tradeoff, which means that more accurate sensors tend to be more expensive [10,11]. However, it is expected that its cost will become progressively lower thanks to recent technological advances [12].

## 2. Related Works

In recent decades, numerous calibration methods for capacitance, TDR, and FDR soil moisture sensors have been carried out using various fluids, soils, and different statistical curve-fitting methods [13].

Capacitive soil moisture sensors operate on the dielectric principle and essentially consist of a pair of electrodes forming a soil capacitor that acts as a dielectric between them [13]. In this sense, dielectric properties are crucial for understanding the behavior of water in soil, in particular soil water content (SWC), as they measure the ability of a material to store an electrical charge and are influenced by water and other minerals in the soil [14]. Many sensors that measure the dielectric constant of the medium yield an indirect signal proportional to the volumetric water content (VWC). This signal manifests as changes in dielectric permittivity such as voltage level (V or mV), which is sampled by a data acquisition device and subsequently processed by software applications [9].

Proper interpretation of the output signal of the sensor requires a calibration procedure to relate it to the soil moisture content, considering factors such as soil texture and structure, temperature, and water salinity [15]; therefore, the direct quantification of soil moisture is not a simple procedure [16]. It has been observed that measurements from dielectric sensors are generally sensitive to salinity, density, mineral/clay content, and soil texture [12,13,14]. Furthermore, an increase in water salinity will result in a higher apparent volumetric water content being measured by a capacitance sensor. In addition, an increase in water salinity increases the apparent volumetric water content measured by a capacitance sensor due to the high electrical conductivity. This represents a challenge for the calibration and use of these sensors in saline soils, especially at low frequency. Recent research proposes improved methods for calculating soil water content and pore water electrical conductivity, including the apparent electrical conductivity of the soil. However, these methods have not yet been tested with low-cost capacitance probes [17].

Most sensors used to measure soil water content at frequencies ranging from kilohertz to gigahertz. To properly interpret how these sensors respond to different frequencies, it is essential to adjust the structural parameters of the model representing Maxwell–Wagner relaxation effects [18]. This effect characterizes the interfacial polarization that occurs between two different materials due to the difference in their dielectric properties, i.e., permittivity and conductivity [10]. Therefore, to achieve higher measurement accuracy, it is necessary to perform a direct calibration between sensor response and moisture content, while considering soil variability, to ensure an accurate prediction of water content [16].

Strategies aimed at reducing irrigation water inputs should be further assessed to determine their potential impact on crop performance and the soil microbial communities that drive critical biogeochemical cycles [19]. Among the soil recovery strategies, organic amendments stand out. Specifically, the intrinsic properties of biochar—such as its high porosity, carbon content, and absorption capacity—can enhance water retention and nutrient availability, thus promoting microbial colonization and improving crop phenological stages [20,21].

It is also important to highlight that measuring electrical conductivity in soil is a foundational aspect of precision agriculture [22]. The main contributions of this paper are as follows:

- Assessment of low-cost soil moisture sensors: this paper evaluates the accuracy and functionality of low-cost soil moisture sensors in sandy loam (SL) soil amended with biochar and fertilizers.
- Sensor calibration of soil biochar mixtures: a calibration for different biochar and fertilizer doses, applied across various soil moisture levels, is used to identify the reliability of soil moisture data under non-standard soil conditions.

The paper is organized as follows: Section 2 reviews related works, providing context and comparing current approaches with the proposed methods. Section 3 covers the materials and methods, with Section 3.1 detailing soil sampling and the soil–biochar mixture process, while Section 3.2 focuses on fertilizer dosage based on potassium (K) and nitrogen (N). Section 3.3 describes the preparation of mixtures incorporating fertilizers and biochar. Section 3.4 provides a description of the sensors used in the study, Section 3.5 details the calibration procedure, and Section 3.6 details the statistical analysis applied to the collected data. Section 4 presents the study results, beginning with Section 4.1, which examines sensor variability. Section 4.2 evaluates sensor performance, and Section 4.3 assesses sensor stability under different conditions. Section 5 analyzes the results and interprets them in the context of existing research, exposes the limitations of this paper and suggests possible future research. Finally, Section 6 concludes the paper, highlighting the main contributions.

## 3. Materials and Methods

This research was conducted in the Laboratory of Soil Physics of the School of Chemistry at the Costa Rican Institute of Technology (ITCR), Cartago, Costa Rica (951026.0900 N, 8354044.3300 W), under stable temperature conditions of 20 °C.

### 3.1. Soil Sampling and Amendment

Soil material was collected from the topsoil (0–30 cm) of an Andisol in localities of the Central Valley of Costa Rica (Table 1). Feedstock biochar was collected from bamboo culms (culms) of *Guadua angustifolia* Kunth pyrolyzed at 400 °C, crushed, and passed through a 3.5 mm sieve.

### 3.2. Fertilizer Dosage Based on K and N

Two types of fertilizers based on potassium (K) and nitrogen (N) were used as separate treatments. A foliar application of soluble powder of K-fertilizer, containing a high concentration of potassium (K) at 53% and phosphorus (P) at 32%, was used along with other nutrients in lower proportions like free amino acids at 3% (L-amino acids) and organic extracts at 2%. Nitrogen (N) was applied in a granulated form, comprising 46% in ureic form (CH_4_N_2_O).

Salinity levels were categorized into three groups: non-saline (NS) at 1.8 dS/m, slightly saline (SS) at 3 dS/m, and moderately saline (MS) at 4 dS/m. Plant tolerance to salinity was assessed based on yield reduction in saline-affected soils relative to yields in non-saline soils, following FAO guidelines [21]. Salinity was measured by means of electrical conductivity (EC) using a water conductivity meter (Hanna Instruments HI98312, Smithfield, RI, USA). Table 2 shows the doses of fertilizer, and the associated electrical conductivity of the soil solution (EC) used in the calibration.

### 3.3. Preparation of Mixtures Incorporating Fertilizers and Biochar

The soil–biochar mixtures were prepared by adding biochar at application rates of 1.5% and 3% (mass percent) to the dry-sieved soil, equivalent to 15.6 and 31.2 ton/ha [23]. Application rates were calculated considering a depth of 15 cm and aiming for the lowest cost possible, as higher biochar contents tend to be rejected by farmers due to their perceived high cost [24]. Treatments were composed of the sandy loam soil (SL) mixed with a fraction of 0, 1.5, or 3% (named B0, B1.5, and B3, respectively) biochar as well as three doses of fertilizer (N or K), the amount of which was based on three salinity levels (NS, SS, and MS) and the mass of soil packed in the pots, as indicated in Table 2. Table 3 presents the array of the 21 treatment combinations. For example, SLB0 corresponds to the treatment with sandy loam soil with no biochar and SLB0K5 belongs to the treatment of sandy loam with no biochar and 5 g of potassium.

Each treatment combination was duplicated and placed in truncated cone pots with a diameter of 27.6 cm at the bottom, 32.0 cm at the top, and a height of 15 cm. Each pot was filled with 7.5 kg of the air-dried soil mixture and poured to obtain a soil bulk density of 1.1 g/cm^3^.

### 3.4. Sensor Description

**Figure 1:**
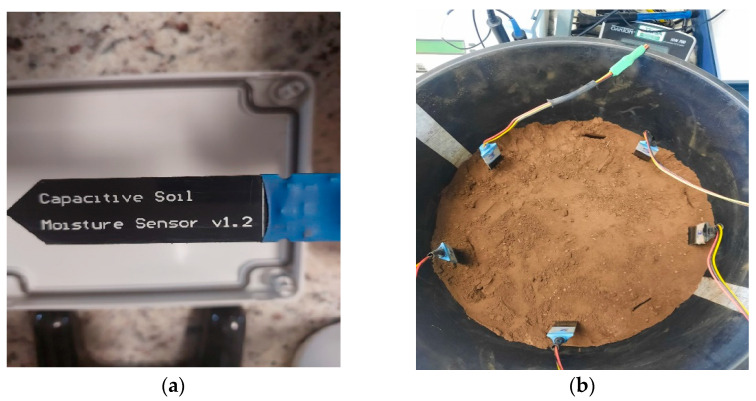
Soil moisture sensor used (**a**) capacitive soil moisture sensor v1.2 and (**b**) set of 5 soil moisture sensors installed for calibration.

The capacitive soil moisture sensors (V1.2) record capacitance variations through a circuit that generates a voltage proportional to the capacitance formed by the sensor upon insertion into the soil. This circuit is integrated into the sensor itself, which produces an output ranging from 0 to 3 V for soil moisture within the 0–100% range. It uses a digital signal with a variable frequency between 260 and 520 Hz depending on the capacitance sensed in response to changes in the soil moisture, which is filtered through an RC circuit to provide a DC output value [25]. Figure 1a illustrates the sensor utilized in this study.

For calibration, five sensors were inserted vertically at 5.2 cm distance from each other, at a depth of 5.5 cm to ensure a good contact between the sensor and the soil (Figure 1b). The sensor output was determined by the relation between the capacitance and the mean apparent volumetric water content measured continuously for 50 min at each soil moisture level, with a one-minute output reading.

### 3.5. Calibration Procedure

The gravimetric soil moisture (W) method was applied for calibration, defined as the water lost by the soil when it dries to constant mass at 105 °C, and it is estimated by Equation (1) [26]:

(1) $$W=\frac{M_{w}-M_{d}}{M_{d}}$$

where $M_{w}$ was the wet soil mass, and $M_{d}$ the dry soil mass.

The drying process was conducted for 24 h at 105 °C to ensure that the soil reached a constant mass. The time required to achieve a constant mass can vary depending on the soil sample, but 24 h was determined to be sufficient for the samples analyzed [27].

The gravimetric method is a direct and highly reliable technique for determining soil moisture, serving as the standard for calibrating soil moisture procedures [24]. Volumetric soil water content was calculated by multiplying gravimetric water content by soil bulk density. Soil moisture data from both replicates were averaged to provide a relative value corresponding to the analog output. A total of six soil water contents were analyzed (0.03, 0.06, 0.16, 0.24, 0.36, and 0.42 cm^3^cm^−3^).

### 3.6. Statistical Analysis

RStudio v. 4.3.1 was used for the statistical analysis, and initially we applied the interquartile range (IQR) statistical method to detect outliers in continuous distribution data. Thereafter, base plots were generated, showing the behavior of the soil moisture measured by the direct method compared to the soil moisture calculated by the sensors, in a time interval of 50 min per soil moisture level.

To ensure the integrity of the results, minimize systematic errors, and determine the calibration errors of the low-cost soil moisture sensor, under identical laboratory conditions, two calibrations were conducted per treatment with a 2.5-week interval between each calibration, according to the process described previously in Section 3.5. The precision of the measuring instruments was verified by obtaining the relative error, following equation (Equation (2)):

(2) $$Relative\;Error\;\%=\frac{Cal\;1-Cal\;2}{Cal\;1}x\;100$$

where Cal 1 indicates the first calibration and Cal 2 indicates the second calibration performed within 2.5 weeks difference.

In the field of precision agriculture, the current strategy is to collect data using sensors, which are then analyzed and classified into specific behavioral patterns using appropriate machine learning models [28]. A useful equation is second-degree polynomial regression, which models the nonlinear relationship between the predictor variables x (sensor output) and the response variable y (soil moisture).

The resulting regression equation was calculated as a function of soil salinity (Equation (3)):

(3) $$y=\beta_{0}+\beta_{1}x+{\beta_{2}x}^{2}+\epsilon$$

where the accuracy of the predictions was evaluated to provide information on the contribution of each sensor to the soil moisture estimation. The variable x represents linear influence, acting as the independent variable in a first-degree model. As x increases, the variable of interest also increases or decreases proportionally. On the other hand, *x*^2^ represents quadratic influence, suggesting a nonlinear relationship between the independent variable and the variable of interest, which may exhibit curvature in its form.

Data splitting involves dividing a dataset into training and testing sets. Models are trained on the training data and tested on the separate testing data to evaluate their performance and prevent overfitting [29].

The database was randomly split before model building, where 80% of the original dataset was used for training and the remaining 20% to evaluate the model performance, where the training data are used to deduce the parameters that described the polynomic relationship between the sensors output value and the moisture level, and the validation data are used to test out the model with data that was not used during the development process, in order to reduce the bias and verify the model performance under new data [30].

The performance of the developed models was assessed based on the goodness of fit ($R^{2}$), the root mean squared error (RMSE), and the percent bias (PBIAS):

(4) $$R^{2}=1-\frac{\sum_{i=1}^{n}{\left(Y_{i}^{obs}-Y_{i}^{pred}\right)}^{2}}{\sum_{i=1}^{n}{\left(Y_{i}^{obs}-\bar{Y^{obs}}\right)}^{2}}$$

(5) $$RMSE=\sqrt{\frac{1}{n}\sum_{i=1}^{n}{\left(Y_{i}^{obs}-Y_{i}^{pred}\right)}^{2}}$$

(6) $$PBIAS=(\frac{\sum_{i=1}^{n}\left(Y^{obs}-Y^{pred}\right)}{\sum_{i=1}^{n}\left(Y^{pred}\right)})\cdot 100$$

where $Y_{i}^{obs}$ and $Y_{i}^{pred}$ were the observed (soil moisture obtained from calibration process) and predicted (value obtained from the developed model) *i*th value, respectively, and $\bar{Y^{obs}}$ was the mean experimental value.

Regression analysis is a common technique used in supervised machine learning, as it is used to predict a continuous value of a target variable based on a set of independent variables or predictor characteristics. The $R^{2}$ coefficient was used to estimate the correlation trend between the observed and predicted data, with values ranging from 0 to 1, where a value closer to 1 indicates a better fit [31].

RMSE values less than half the standard deviation of the observed values are considered good predictors and is optimal for normal (Gaussian) errors [32]. PBIAS was employed to assess the tendency of the predicted data to be either larger or smaller than their observed counterparts, with optimal PBIAS values being equal to 0.0. Negative values indicate under-estimation bias while positive values indicate overestimation. In addition, low-magnitude values suggest an accurate model simulation [33,34].

## 4. Results

### 4.1. Sensor Variability

**Figure 2:**
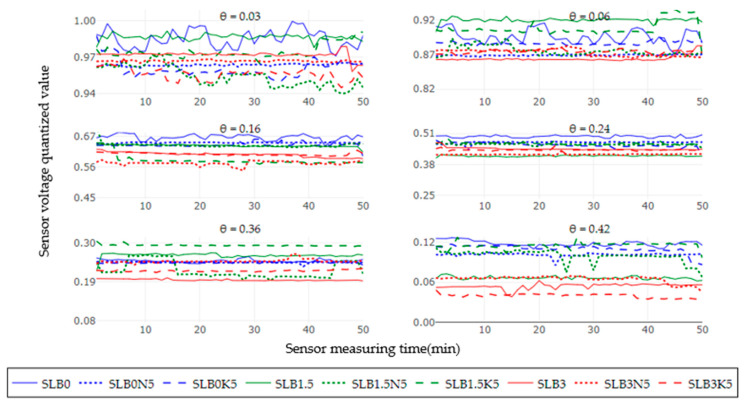
Sensor output performance over time under non-saline (NS) conditions for six soil moisture levels.

**Figure 3:**
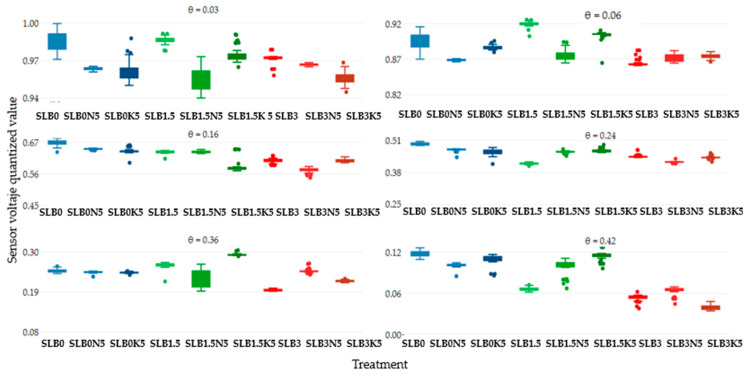
Sensor output dispersion per treatment under non-saline (NS) conditions for six soil moisture levels.

Figure 2 represents the response of the average of five soil moisture sensors for the treatments SLB0, SLB1.5, and SLB3 in the NS scenario with 5 g of fertilizer N and K, giving 50 readings for each soil moisture level, counting for a total of 300 measurements. The treatment SL (continuous blue line) was used as a control to observe variations in the sensor voltage output concerning the effect of the amendments and the NS salinity level. As soil moisture increases, the sensor voltage output decreases, with a general output voltage range between 0.2 and 0.3. Although the voltage output range is small, a consistent trend was observed, showing higher voltage for control treatments without biochar (B0) and with 1.5% biochar (B1.5) compared to treatments with 3% biochar (B3), regardless of the fertilizer used, which is confirmed by Figure 3. Additionally, it was noted that the voltage output tends to be unstable for most of the treatments. Except for SLB0, no clear stability trend can be identified, and outliers are present for all moisture levels.

**Figure 4:**
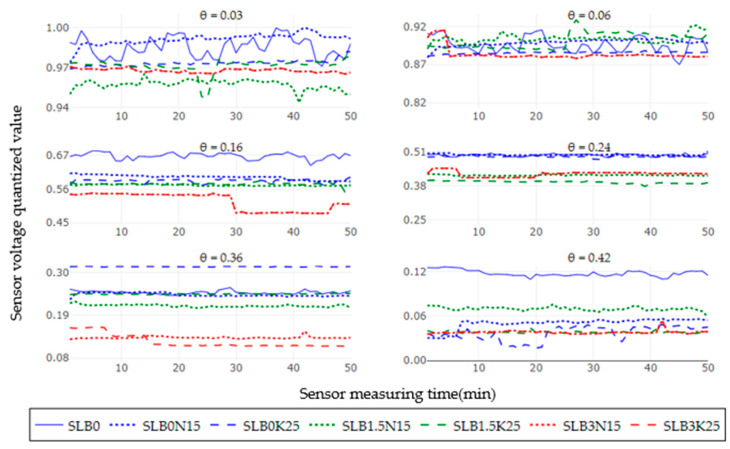
Sensor output performance over time under slightly saline (SS) conditions for different soil moisture levels.

**Figure 5:**
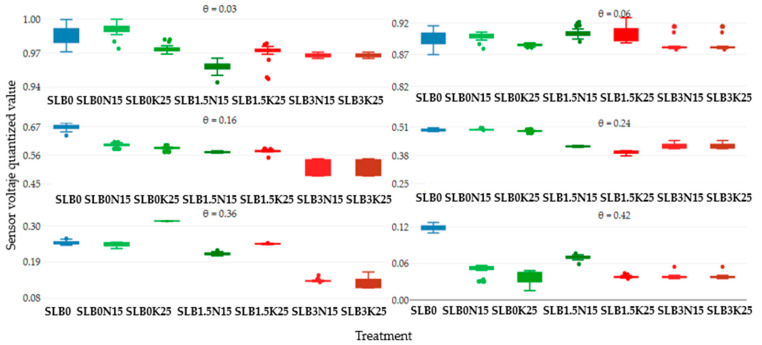
Sensor output dispersion per treatment under slightly saline (SS) conditions for different soil moisture levels.

Figure 4 illustrates the sensor voltage output at six moisture levels for the treatments SLB0, SLB1.5, and SLB3 under the SS scenario with 15 g N and 25 g K fertilizer. In comparison to the NS scenario, all treatments generally show lower sensor voltage outputs relative to the SLB0 (control), particularly in the very dry and saturated soil condition. For the NS scenario, treatments with 3% biochar (B3) exhibit a more consistently low voltage output, regardless of soil moisture and salinity levels. Unstable readings are observed up to a soil moisture level of 0.16 cm^3^cm^−3^ for most treatments, except for SLB0. As in the NS scenario, treatments without biochar and the ones with 1.5% of biochar usually had a higher voltage than the ones with 3% biochar, as shown in Figure 5.

**Figure 6:**
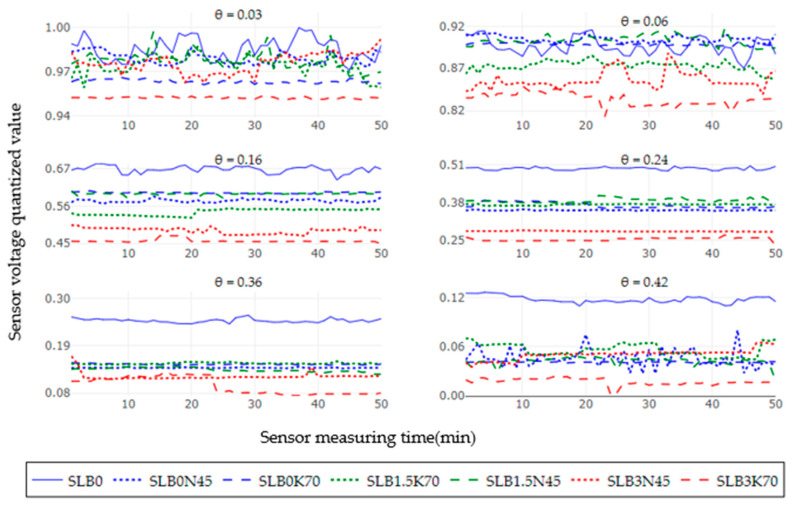
Sensor output performance over time under moderately saline (MS) conditions for six soil moisture levels.

**Figure 7:**
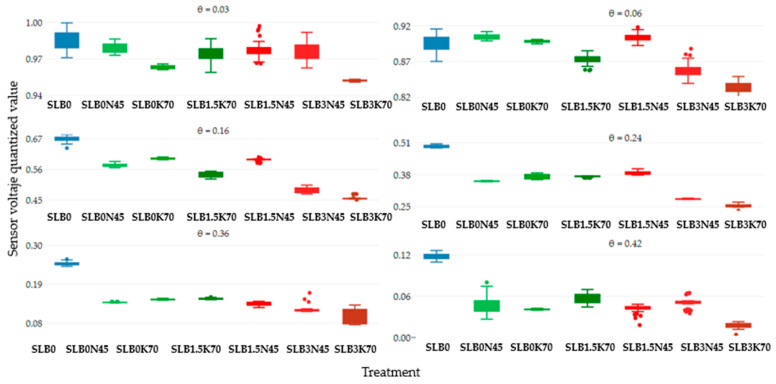
Sensor output dispersion per treatment under moderately saline (MS) conditions for six soil moisture levels.

Figure 6 illustrates the sensor voltage output for the six soil moisture levels across the treatments SLB0, SLB1.5, and SLB3 under the MS scenario with 45 g N and 70 g K fertilizer. Similar to the SS scenario, all treatments exhibit lower voltage outputs compared to control treatment SLB0. As moisture increases, most treatments show more stable readings, although an oscillation pattern is noted for SLB0N45 and SLB1.5K70 at a soil water content of 0.42 cm^3^cm^−3^, and for the same moisture level, in Figure 7 those treatments are the ones with more dispersion (bigger boxplot), even though SLB1.5N45 and SLB3N45 are the ones with more outliers.

### 4.2. Sensor Performance

**Figure 8:**
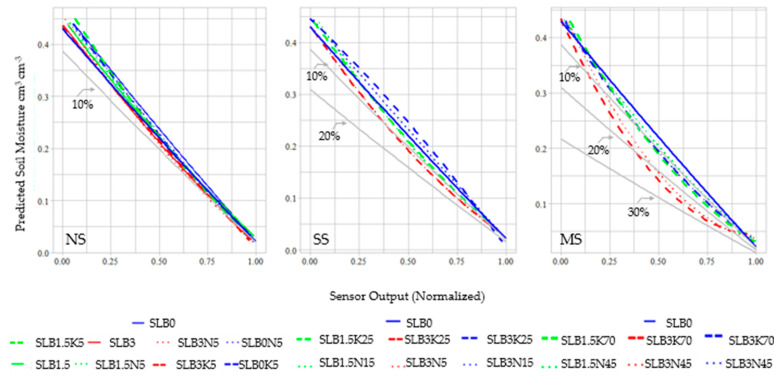
Calibration curves for all treatments under the NS, SS, and MS scenarios. The blue continuous line indicates the calibration curve for SLB0 without fertilizer and the gray lines represent the error deviation of 10%, 20%, and 30% with respect to SLB0.

Figure 8 shows calibration curves for all treatments under the salinity scenarios: NS (no salinity), SS (slight salinity), and MS (moderate salinity). Higher salinity levels and increased biochar addition correlate with greater calibration errors. In the NS scenario, most calibrated soil moisture values exhibit errors below 10%, except for SLB3K5, which shows a slight increase in error as the soil dries. Under the SS scenario, most treatments remain within a 10% range, except for SLB3K25. However, the deviation degree seems related to biochar addition and the soil water content. SLB1.5 treatment shows a deviation close to 10% at moisture levels above 0.30 cm^3^cm^−3^, while SLB3 treatment started with a 20% error in dry conditions, decreasing to about 10% at saturated levels over 0.35 cm^3^cm^−3^. In the MS scenario, all treatments have calibration errors greater than 10% under low water content (dry conditions), with errors decreasing as water content rises, particularly in SLB3 treatments. A threshold of 0.35 cm^3^cm^−3^ (very wet conditions) was noted, where calibration errors dropped below 20% for SLB3 and below 10% for SLB1.5 and SLB0 treatments, respectively. Furthermore, the calibration curves for SLB3 treatments at MS levels exhibit a polynomial shape, while other curves appear linear.

The calibration curves provide the values for the intercept, x and $x^{2}$, as shown in Table 4. Notably, all treatments share the same intercept value, indicating that in the regression model, when all predictor variables are zero, the expected response variable is approximately 0.216 for each treatment. This suggest that all treatments start from a similar baseline regarding the response variable, accounting for the effects of predictor variables, specifically salinity. Because of the nature of the developed model, the coefficients do not correspond to the standard a, b, and c values of a typical quadratic equation ($ax^{2}+bx+c$); instead, they undergo a polynomial transformation using the poly() function in R.

In the regression analysis, the fitted model between the actual and measured soil water content, shown in Table 5, showed $R^{2}$ values close to 1, indicating a strong correlation between the data. Specifically, all SLB0 and SLB1.5 (from MS) treatments achieved an $R^{2}$ value equal 0.999. The RMSE values were generally low, averaging about half the standard deviation (SD) of the measured data. The treatments with the lowest RMSE values were SLB0K5, SLB1.5K70, and SLB1.5N45, each with an RMSE of 0.004 compared to an overall SD of 0.14. PBIAS exhibited considerable variability, reflecting the different levels of bias in the models. Treatments such as SLB0K5, SLB1.5, SLB1.5N5, and SLB3K70 resulted in negative PBIAS, indicating an underestimation of the actual water content. Conversely, treatments like SLB0N5, SLB1.5K70, and SLB3K25 showed overestimation, as reflected by positive PBIAS.

### 4.3. Sensor Stability

**Figure 9:**
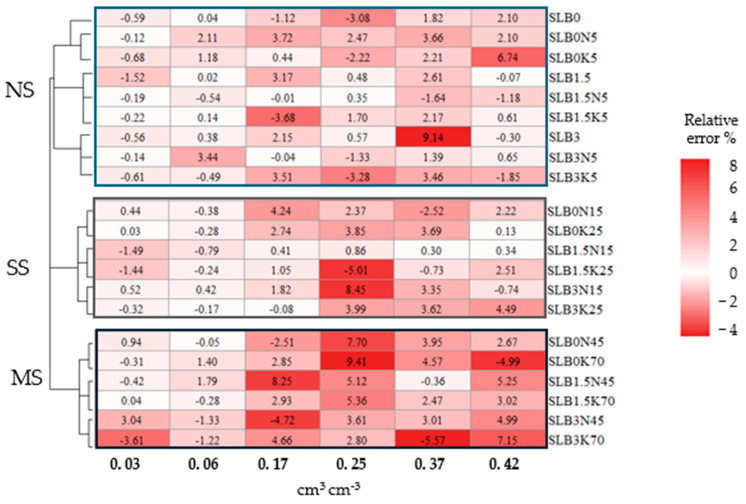
Performance of the soil moisture sensor through errors in treatment calibrations in a 2-week interval.

Figure 9 depicts sensor performance based on two calibrations per treatment over a 2.5-week interval. The relative error ranged from −4 to 8%. As soil moisture falls below 0.06 cm^3^cm^−3^, fluctuations in sensor response between the two calibrations decrease, particularly for NS and SS. Although the relative error increases for the NS and SS scenarios at soil moisture levels above 0.06 cm^3^cm^−3^, it generally remains lower than in the MS scenario.

**Figure 10:**
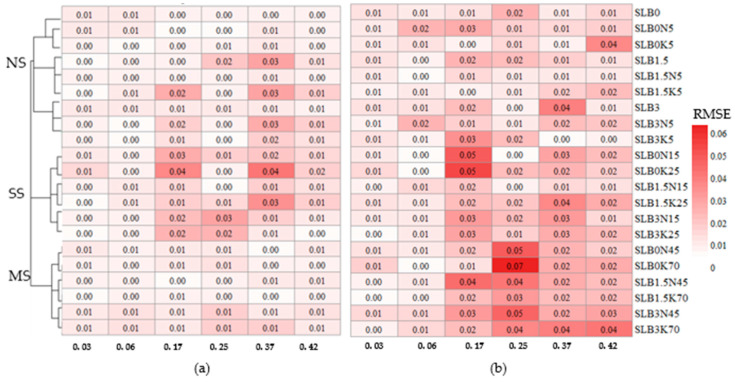
Heat map of the RMSE pattern for all treatments for (**a**) first calibration and (**b**) second calibration after a 2-week interval.

Figure 10 displays two heatmaps illustrating error distributions for various treatments obtained from two calibrations over a 2.5-week interval. This analysis assessing the model’s performance predicts soil moisture with different salinity levels and biochar fractions. The first calibration (Figure 10a) reveals generally low RMSE values (<0.04 cm^3^cm^−3^) across all treatments, regardless of salinity levels. However, in the second calibration (Figure 10b), a slight increase in RMSE was noted in the MS scenarios, particularly at soil moisture levels exceeding 0.17 cm^3^cm^−3^, with a notable increase in the SLB3K70 treatment.

## 5. Discussion

Soil water content and salinity impact low-capacitive soil moisture sensors. Calibration curves for biochar-amended soils and varying high salinity levels are inadequately explained by simple regression models, as noted in Zawilski et al. [35]. This complexity arises from uneven water content distribution in high-adsorption clay soils and is comparable to the biochar material, like our biochar case study. The heterogenous amendments may affect the Maxwell–Wagner polarization, influencing dielectric response based on phase composition and geometry [35], leading to higher permittivity at low frequencies [36]. The nonlinear calibration curves indicate high sensitivity and the uncertainty of sensors, particularly at elevated water content, aligning with findings by Peddinti et al. [13], who emphasized the water content’s significant influence on capacitive and conductive soil behavior. Whereas salinity influences the soil conductive behavior alone. Soil water forms dipoles, increasing dielectric permittivity and energy storage, while the dissipation factor decreases with higher moisture, indicating reduced energy loss [35,37]. At moisture levels above 0.24 cm^3^cm^−3^, especially in lower and medium saline conditions (SS and MS), significant variability between treatments was evident, linked to soil capacitance changes affecting sensor performance. Therefore, specific calibrations for varying moisture conditions are essential for accurately estimating soil water content with low-cost sensors.

The dielectric constant of soil capacitive sensors is strongly influenced by soil water content and composition [37]. Since calibration for all treatments was carried out at the same water content levels, it is assumed that the output signal was primarily affected by soil composition, particularly the addition of biochar. Properties related to soil texture and structure—such as organic matter, pore size distribution, clay content, bulk density, temperature, and frequency—impact dielectric properties, dielectric dispersion, bound water relaxation, and interphase conditions [18,37], ultimately affecting the accuracy of soil water content measurement [38].

Biochar’s composition, porosity, and high surface area modify soil physicochemical properties, thus affecting the soil dielectric constant. Its moisture retention capacity likely increases the adsorbed water in the amendment, leading to a higher effective dielectric constant detected by the sensor, and consequently reducing the output voltage. Biochar amendment porosity composition is another mechanism that can influence the sensor response. The porosity of biochar amendments also influences sensor response. Variations in particle size affect the porosity and bulk density of the mixtures [39]. Since volumetric soil water content depends on bulk density, the water content in amended soils may differ from that in unamended soils at specific moisture levels, impacting the number of water molecules per unit volume (volumetric water content). This influences the dielectric contribution of water rather than its gravimetric water content [40]. This aligns with findings from Zawilski et al. [35], which noted that soil compaction affects dielectric properties, as soil bulk density influences particle spacing.

Capacitive sensors are influenced by dissolved salts, which alter the medium’s chemical properties, and their accuracy is closely related to the working frequency [41]. Measured capacitance increases with rising electrical conductivity (EC) [15], while higher frequencies reduce the impact of EC [39]. Increased porosity, associated with greater solute retention, decreases the dielectric layer’s modulus of elasticity, resulting in higher capacitance within the sensor. Consequently, sensor sensitivity [42] improves under higher salinity conditions, with predicted soil moisture for biochar amendments diverging by up to 30% from the unamended SLB0 treatment. Changes in capacitive sensors during calibrations with salinity highlight the need for specific calibration adjustments for each scenario. This necessity is underscored by the significant variability observed among treatments at elevated salinity and moisture levels, as indicated by Chen et al. [19].

The proposed model maintained a stable level of predictive error across all the treatments, indicating consistent error dispersion. The small variation in standard deviation indicates that the model’s accuracy remains largely unaffected by different treatment conditions, reinforcing its overall reliability. The similar error distribution across treatments suggests low error dispersion. Comparatively RMSE and PBIAS show greater a variability than standard deviation among treatments, and the overall error variability remains consistently low. However, observations over time reveal that sensor behavior changes, highlighting the need for ongoing monitoring and frequent recalibration.

In this context, the availability of cost-effective devices and accurate methods emerges as a crucial factor for promoting the adoption of smart systems in agriculture. These systems have the potential to significantly reduce the environmental impact of industrial crops, as highlighted by Bertocco et al. in [43]. However, the effective implementation of these systems requires a comprehensive network of connected monitoring devices capable of covering extensive cultivation areas and controlled environments such as greenhouses. This necessity underscores the importance of continuing to develop technologies that enable efficient and economical monitoring and recalibration.

Nonetheless, water management in agriculture and progress towards sustainability still require further research [44,45] as numerous factors affect volumetric water content measurements [46].

### 5.1. Limitations of the Study

A primary limitation of the proposed method is its dependence on soil-specific data, which necessitate an initial setup and calibration phase involving the collection of additional data related to the soil type. However, the proposed methods can be adapted to various soil types.

A notable challenge is the need for regular sensor recalibration and precise data estimation. Simply averaging sensor readings may not suffice for obtaining robust results, which is a well-known issue in data fusion; however, other filtering and adapting techniques can be explored to relax this calibration requirement and the impact of deficient measurements.

### 5.2. Future Work and Outlook

To improve the accuracy and robustness of sensor calibration and data estimation, advanced data fusion techniques such as Kalman filters or Bayesian algorithms could be incorporated as future work. These methods can adjust calibration errors and optimize predictions over time, providing more reliable estimates under various conditions. For instance, the data assimilation method Ensemble Kalman Filter (EnKF) was used by Zhang et al. [47] for comparison with the Local Error-Subspace Transform Kalman Filter (LESTKF). EnKF formalizes the estimation of model errors by assuming that the ensemble mean represents the “truth” and calculating the variance of the differences between each ensemble member and the ensemble mean.

Implementing calibration protocols that include repeated testing and periodic adjustments could ensure greater consistency and accuracy in measurements over time. The expansion of sensor calibration protocols could include the development and implementation of more sophisticated calibration protocols to address the limitations of current methods, ensuring more reliable sensor performance across different soil conditions.

The inclusion of additional environmental variables constitutes another future exploration area for the integration of additional environmental factors, such as temperature and soil composition, to improve the accuracy of moisture predictions and provide a more comprehensive understanding of soil conditions. Conducting repeated measurements under different environmental conditions will allow for the validation of sensor accuracy and the adjustment of models to better reflect environmental variations.

Finally, the exploration of alternative sensors could expand the portfolio of possible solutions and scenarios over time, which would allow for a comparison of performance and an identification of the most suitable option in terms of cost and effectiveness.

## 6. Conclusions

The importance of calibrating the sensors for the specific application is evident, as significant changes can occur depending on the soil mixture used. The reliability of capacitive sensors for estimating water content is influenced by soil matrix composition, electrical conductivity, and dielectric properties, making them highly sensitive to soil salinity; therefore, their accuracy requires frequent monitoring.

Establishing the correlation between biochar amendments and dielectric properties poses a challenge in calibrating and validating dielectric sensors for soil water content monitoring. Furthermore, existing studies lack a comprehensive understanding of how volumetric conditions in biochar soil mixtures influence sensor performance.

To fully understand the effect of biochar amendments on low-frequency capacitive soil moisture, further research on the dielectric properties of biochar materials and their impact on agricultural amendments is essential. Low-cost calibration soil moisture sensors require more than simple regression models to accurately estimate the complexities of soil properties related to agricultural management practices like fertilization and biochar application.

Despite the high cost of advanced auto-calibrated sensors for multi-point acquisition in precision agriculture, low-cost sensors are a practical alternative. Their limitations can be mitigated through effective calibration methodologies and data fusion techniques, provided that the underlying physics are well understood and accurately modeled.

## Figures and Tables

**Table 1:** Physical and chemical characterization of the soil material utilized for the calibrations.

Soil Physical Properties			Chemical Soil Properties										
Soil texture (%)			pH	cmol(+)/L					%	mg/L		dS/m	Ratio
Clay	Sand	Slit	H_2_O	Acidity	Ca	Mg	K	ECEC	AS	P	Mn	CE	C/N
24.1	63.6	12.3	6.6	0.1	8.31	3.19	0.94	12.54	0.8	116	8	1.79	8.9

AS%: acid saturation percentage; ECEC: effective cation exchange capacity; EC: electrical conductivity; C/N: carbon to nitrogen ratio.

**Table 2:** Fertilizer dosage based on K and N and its effect on soil solution electrical conductivity (EC) used during calibration for treatments categorized into three salinity levels: NS, SS, and MS.

Doses(g)/EC(dS/m)			
Fertilizer	Non Saline (NS)	Slightly Saline (SS)	Moderately Saline (MS)
**K**	0.76/1.8	0.23/3	6/4
**N**	0.70/1.8	0.38/3	10/4

**Table 3:** Treatment combination of soil, biochar, and salinity levels.

Treatment	Non-Saline NS (g)	Sightly Saline SS (g)	Moderately Saline MS (g)	Biochar (%)	No. of Treatments
SLB0	-	-	-	0	1
SLB1.5	-	-	-	1.5	1
SLB3	-	-	-	3	1
SL0N	5	15	45	0	3
SLB1.5N	5	15	45	1.5	3
SLB3N	5	15	45	3	3
SL0K	5	25	70	0	3
SLB1.5K	5	25	70	1.5	3
SLB3K	5	25	70	3	3

SL: sandy loam soil; 0, 1.5 and 3: biochar dose; N: nitrogen fertilizer; K: potassium fertilzer.

**Table 4:** Polynomial equation coefficients obtained from fitted calibration curves for all treatments under the NS, SS, and MS scenarios.

Treatment	Polynomial Coefficient		
	Intercept	x	x ^2^
SLB0	0.216	−2.248	0.031
SLB0N5	0.216	−2.248	0.035
SLB0K5	0.216	−2.247	0.079
SLB1.5	0.216	−2.237	0.096
SLB1.5N5	0.216	−2.247	0.049
SLB1.5K5	0.216	−2.228	0.164
SLB3	0.216	−2.245	0.089
SLB3N5	0.216	−2.234	0.149
SLB3K5	0.216	−2.245	0.037
SLB0N15	0.216	−2.237	0.026
SLB0K25	0.216	−2.218	−0.069
SLB1.5N15	0.216	−2.238	0.183
SLB1.5K25	0.216	−2.230	0.144
SLB3N15	0.216	−2.229	0.211
SLB3K25	0.216	−2.228	0.204
SLB0N45	0.216	−2.234	0.251
SLB0K70	0.216	−2.241	0.177
SLB1.5K70	0.216	−2.231	0.284
SLB1.5N45	0.216	−2.242	0.172
SLB3N45	0.216	−2.200	0.449
SLB3K70	0.216	−2.199	0.450

**Table 5:** Calibration errors goodness of fit ($R^{2}$), root mean squared error (RMSE), percent bias (PBIAS), and standard deviation (SD) obtained for each of the models for all treatments.

Treatment		Error		
	$R^{2}$	RMSE	PBIAS	SD
SLB0	0.999	0.005	0.127	0.146
SLB0N5	0.999	0.006	0.230	0.146
SLB0K5	0.999	0.004	−0.265	0.146
SLB1.5	0.990	0.015	−0.497	0.145
SLB1.5N5	0.997	0.008	−0.710	0.145
SLB1.5K5	0.986	0.017	−0.146	0.145
SLB3	0.998	0.007	0.115	0.146
SLB3N5	0.990	0.014	−0.045	0.145
SLB3K5	0.996	0.009	−0.021	0.146
SLB0N15	0.990	0.015	0.014	0.145
SLB0K25	0.971	0.025	0.070	0.144
SLB1.5N15	0.996	0.009	−0.436	0.146
SLB1.5K25	0.986	0.017	−0.283	0.145
SLB3N15	0.993	0.012	−0.210	0.146
SLB3K25	0.992	0.013	0.084	0.147
SLB0N45	0.998	0.006	−0.311	0.146
SLB0K70	0.999	0.005	−0.253	0.147
SLB1.5K70	0.999	0.004	0.226	0.148
SLB1.5N45	0.999	0.004	−0.017	0.146
SLB3N45	0.996	0.009	−0.240	0.147
SLB3K70	0.995	0.011	−0.939	0.146

## Data Availability

Authors can share data on request addressed to kvillagra@tec.ac.cr.
